# Visceral Leishmaniasis in Rural Bihar, India

**DOI:** 10.3201/eid1810.111083

**Published:** 2012-10

**Authors:** Epco Hasker, Shri Prakash Singh, Paritosh Malaviya, Albert Picado, Kamlesh Gidwani, Rudra Pratap Singh, Joris Menten, Marleen Boelaert, Shyam Sundar

**Affiliations:** Institute of Tropical Medicine, Antwerp, Belgium (E. Hasker, A. Picado, J. Menten, M. Boelaert);; and Banaras Hindu University, Varanasi, India (S.P. Singh, P. Malaviya, K. Gidwani, R.P. Singh, S. Sundar)

**Keywords:** leishmaniasis, visceral, risk factors, epidemiology, analysis, multilevel, parasites, India, Leishmania, vector-borne infections

## Abstract

To identify factors associated with incidence of visceral leishmaniasis (VL), we surveyed 13,416 households in Bihar State, India. VL was associated with socioeconomic status, type of housing, and belonging to the Musahar caste. Annual coverage of indoor residual insecticide spraying was 12%. Increasing such spraying can greatly contribute to VL control.

Visceral leishmaniasis (VL), a vector-borne parasitic disease caused by several *Leishmania* spp., is nearly always fatal if left untreated (*1*,*2*). The clinical syndrome is characterized by fever, weight loss, splenomegaly, hepatomegaly, and anemia. The disease is endemic in >60 countries, but 90% of all reported cases occur in just 5 countries: Bangladesh, Brazil, India, Nepal, and Sudan (*3*). On the Indian subcontinent, the disease is assumed to be an anthroponosis; the vector is a sand fly, *Phlebotomus argentipes*. Approximately 200 million persons on the Indian subcontinent are at risk for VL, and the annual incidence is ≈420,000 cases (*4*). The disease affects mainly poor rural communities; ≈80% of all cases in the region are reported from the state of Bihar in India (*4*).

Earlier studies on the Indian subcontinent have identified several risk factors for VL (*5*–*11*). At times, findings between studies have been conflicting, particularly in relation to the role of domestic animals (*7*). The use of bed nets was found to be protective in some studies (*2*,*5*), but this conclusion could not be confirmed in a recent cluster-randomized trial (*12*). Many of the earlier studies were conducted on fairly small populations, usually 1 or 2 villages (*5*,*6*,*8*,*9*); confounding by socioeconomic status was controlled to a varying extent. Most studies were conducted in high-incidence villages or in villages in which a recent outbreak had occurred. Because VL has a strongly clustered distribution, understanding the reasons behind widely varying incidence levels among villages and hamlets could also be useful. We therefore studied factors associated with VL in an area made up of villages with variable levels of VL incidence and constructed an asset index to control for confounding by socioeconomic status.

## The Study

The study area is a geographically continuous area comprising 50 villages in the Muzaffarpur District of Bihar State, India, a district where VL is highly endemic. The 50 villages can be further subdivided into 200 hamlets, also known as *tolla*. We conducted 3 annual surveys in September and October of 2008, 2009, and 2010. In each survey, we visited all households in the study area, collected demographic information, and asked whether the house had been covered by indoor residual insecticide spraying in the year preceding the survey. In each survey, we also collected information about VL in the household since the previous survey. For the first survey, we used a recall period of 1.5 years. A case of VL was defined as the combination of a clinical history typical for VL (fever of >2 weeks’ duration, lack of response to antimalarial drug treatment); a positive result by the rK39 rapid diagnostic test; and a good response to specific VL treatment, with or without confirmation of parasites. Each case reported was verified from medical records by a study physician. At the time of the second survey in 2009, we also collected information about assets owned by each household, including domestic animals, and we recorded characteristics of the structure of the house and the surrounding vegetation. Information about assets owned (other than domestic animals) was used to subdivide the study population into 5 quintiles of socioeconomic status. To study potential associations with household environment, remote sensing (15-m resolution ASTER images), and ground data were combined to derive a map of the study area showing 7 types of land cover. Data were analyzed in a binomial multilevel model with *tolla* as a random effect.

We enrolled a study population of 81,210 persons, divided over 13,416 households (92% of all households in the study area). During the study period, we registered 207 VL cases, equivalent to an average annual incidence of 72.8/100,000 population. Cases were strongly clustered at the *tolla* level, with an intraclass correlation of 32%. None of the types of land cover was significantly associated with disease. With the villages spread out along roads and farming land split up into small plots, the environment experienced by persons from different villages or from different locations did not vary greatly.

VL was strongly associated with age; the odds of having VL was lowest for children <5 years of age and highest for children 5–14 years of age (odds ratio [OR] 2.5, 95% CI 1.5–4.0). Higher socioeconomic status was associated with reduced risk; comparing the wealthiest to the poorest quintile, we observed an OR of 0.5 (95% CI 0.3−1.0). Having at least 1 bed net per 3 household members was protective on univariate analysis, but the effect was weaker and no longer statistically significant when we controlled for confounding (OR 0.8, 95% CI 0.5–1.3). This finding can be explained by a strong association between socioeconomic status and ownership of bed nets.

Of ownership of all the domestic animals investigated, only ownership of goats was weakly, but significantly, associated with VL (OR 1.4, 95% CI 1.0–1.8). Other factors at household level that were statistically significant in multivariate analysis were the following: belonging to the Musahar caste (OR 2.9, 95% CI 1.3–6.8); presence of a bamboo tree (OR 1.5, 95% CI 1.2–2.0); and type of walls (OR 1.8, 95% CI 1.0–3.3 for unplastered brick walls and OR 2.5, 95% CI 1.3–4.6 for thatched walls, with plastered brick walls as reference for both). (Table) Thatched walls and presence of bamboo trees are likely to provide favorable breeding conditions for the sand fly vector (*13*).

**Table:** Factors associated with visceral leishmaniasis, Bihar State, India, 2008–2010

Factor	No. (%) participants		Odds ratio*
	Total, N = 81,210	Case-patients, n = 207	
Demographic characteristic			
Mushahar caste	1,980 (2.4)	32 (15.5)	2.9 (1.3–6.8)
Age group, y			
0–4	12,787 (15.8)	20 (9.7)	Referent
5–14	21,020 (25.9)	79 (38.2)	2.5 (1.5–4.0)
15–24	14,282 (17.6)	33 (15.9)	1.7 (1.0–3.0)
25–34	10,993 (13.5)	31 (15.0)	2.0 (1.1–3.5)
35–44	8,462 (10.4)	23 (11.1)	1.9 (1.1–3.6)
>45	13,666 (16.8)	21 (10.1)	1.2 (0.6–2.2)
Socioeconomic status, by assets index level			
Level 1, poorest	16,515 (20.3)	70 (33.8)	Referent
Level 2	16,094 (19.8)	58 (28.0)	0.9 (0.6–1.3)
Level 3	16,124 (19.8)	31 (15.0)	0.7 (0.5–1.1)
Level 4	16,256 (20.0)	35 (16.9)	0.9 (0.6–1.4)
Level 5	16,221 (20.0)	13 (6.3)	0.5 (0.3–1.0)
Other			
Ownership of goats	25,703 (31.7)	86 (41.6)	1.4 (1.0–1.8)
Bamboo tree at <10 m	31,554 (38.9)	86 (41.6)	1.5 (1.1–2.0)
Type of walls			
Brick, plastered	19,169 (23.6)	15 (7.3)	Referent
Brick, unplastered	35,401 (43.6)	96 (46.4)	1.8 (1.0– 3.3)
Thatched	26,640 (32.8)	96 (46.4)	2.5 (1.3– 4.6)

*Based on multivariate model with *tolla* of residence as random effect.

Indoor residual insecticide spraying coverage was poor. In 2009 (the last year for which data were collected for the full year), only 12% of all households had reportedly been sprayed at least once.

## Conclusions

In this large cohort study, controlled for potential confounding by socioeconomic status and other contextual factors, we identified several factors associated with VL. Ownership of goats and presence of bamboo trees near the house are risk factors, but are not strong enough to warrant specific interventions. Poor housing is a stronger risk factor; thus, housing plans launched by the Indian government may positively affect control of VL. Persons in the Musahar caste were at increased risk; they made up 2.4% of the study population but had >15% of VL cases. The Musahars are known to be among the poorest of the poor, but even after we controlled for confounding by socioeconomic status, the association remained statistically significant. Some residual confounding cannot be ruled out, but other factors probably play a role. One such factor could be long delays in seeking health care by Musahars, which was documented in another recent study (*14*). When devising improved VL control strategies, it would certainly be justified to pay special attention to the hamlets inhabited by the Musahar caste. Overall, however, the most benefit can be expected from strengthening vector control efforts. In the 1960s, as a byproduct of intensive indoor residual insecticide spraying for malaria eradication campaigns, VL was all but eliminated from the area, and biannual indoor residual insecticide spraying is one of the cornerstones of the regional VL elimination strategy (*15*). Thus, it defies imagination that in this highly VL-endemic area, the annual indoor residual insecticide spraying coverage can be as low as 12%.
